# Risk factors for refracture after plate removal for midshaft clavicle fracture after bone union

**DOI:** 10.1186/s13018-019-1516-z

**Published:** 2019-12-21

**Authors:** Shang-Wen Tsai, Hsuan-Hsiao Ma, Fang-Wei Hsu, Te-Feng Arthur Chou, Kun-Hui Chen, Chao-Ching Chiang, Wei-Ming Chen

**Affiliations:** 1 Department of Orthopaedics and Traumatology, Taipei Veterans General Hospital, No. 201, Sec 2, Shi-Pai Road, Taipei, 112 Taiwan; 2 Department of Orthopaedics, School of Medicine, National Yang-Ming University, Taipei, Taiwan; 3 Department of Orthopaedics, Kuang-Tien General Hospital, Taichung, Taiwan

**Keywords:** Clavicle, Implant removal, Midshaft, Refracture, Risk factor

## Abstract

**Background:**

Open reduction and internal fixation (ORIF) with plates and screws is one of the treatment options for clavicle fractures. However, an additional operation for implant removal after union of the fracture is commonly performed due to a high incidence of hardware irritation. Despite union of the fracture, a subsequent refracture might occur after removal of the implant which requires additional surgeries for fixation. This study aims to determine the risk factors associated with refracture of the clavicle after hardware removal.

**Methods:**

We retrospectively reviewed the medical records of 278 patients that were diagnosed with a midshaft clavicle fracture (male 190; female 88) that had (1) undergone ORIF of the clavicle with plates and (2) received a second operation for removal of hardware after solid union of the fracture between 2010 and 2017. Their mean age was 40.1 ± 15.1 years, and mean interval from fixation to plate removal was 12.5 ± 7.5 months. The patients were then divided into two groups based on the presence of refracture (*n* = 20) or without refracture (*n* = 258). We analyzed patient demographics, interval between fixation and implant removal, fracture classification (AO/OTA, Robinson), fixation device, whether wires or interfragmentary screws were used, clavicular length, and bone diameter at the fracture site.

**Results:**

The overall refracture rate was 7.2%, and the mean interval between plate removal and refracture was 23.9 days. A multivariate analysis showed that female (adjusted odds ratio [aOR] 4.74; 95% CI 1.6–14.1) and body mass index [BMI] (for every 1-unit decrease, aOR 1.25; 95% CI 1.06–1.48) were risk factors for refracture. In women, BMI was the only risk factor. The optimal BMI cutoff value was 22.73. In a female patient with a lower BMI, the refracture rate was 29.8%.

**Conclusions:**

There are no significant radiographic parameters associated with refracture. Routine plate removal in a female patient with a low BMI after bony union of a midshaft clavicle fracture is not recommended because of a high refracture rate.

## Introduction

Although there is still a debate between conservative treatment and surgical treatment of displaced midshaft clavicle fracture, operative treatment for displaced midshaft clavicle fractures is associated with a lower incidence of nonunion, symptomatic malunion, and improved short-term functional outcome and patient satisfaction compared with that of nonoperative treatment [1–3]. Regardless of the initial treatment method (operative or nonopeartive), a subsequent operation is still frequently required to achieve the best overall outcome [4]. Isolated implant removal is the most common type of reoperation in patients who have undergone operative treatment with an incidence of 8–50% [5–8]. Refracture after implant removal has been reported in some studies, with an incidence rate ranging from 1 to 5% [9–11]. Despite a relatively high incidence of refracture after removal of hardware, there is minimal literature discussing the risk factors associated with this condition. This study was aimed to identify the risk factors that may result in refracture of the clavicle. We hypothesize that refracture is multifactorial and associated with preoperative, intraoperative, and postoperative factors.

## Materials and methods

We retrospectively reviewed the medical records of 406 patients who had (1) undergone ORIF of the clavicle and (2) received a second operation for removal of hardware after solid union of the fracture between 2010 and 2017. All of the patients that underwent a second operation for removal of hardware were due to hardware irritation leading to patient discomfort. This study was approved by our hospital’s institutional review board (IRB #: 2018-04-004AC).

**Fig. 1:**
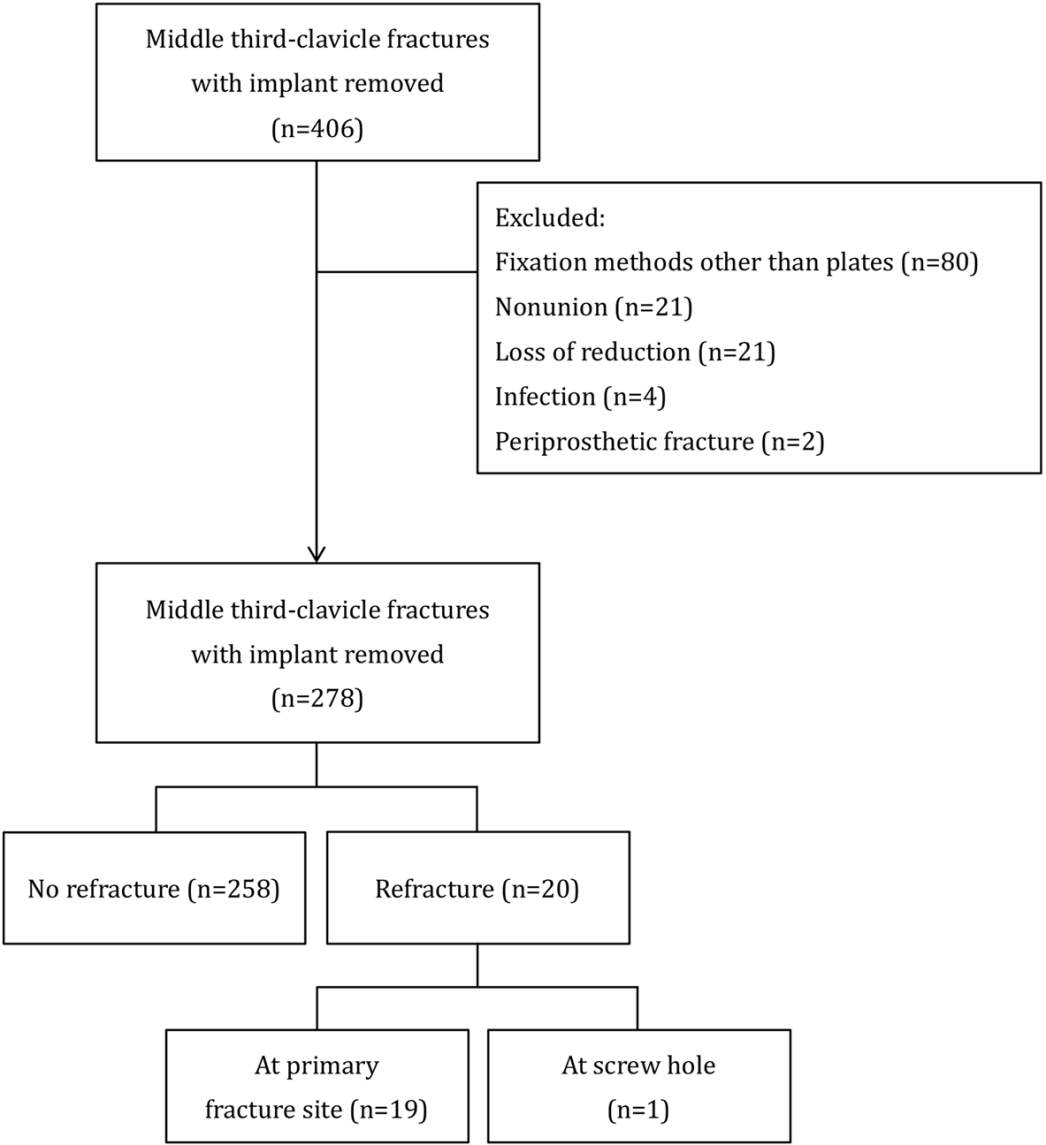
Consort flow diagram

Patients with a midshaft clavicle fracture surgically treated with plate fixation, with radiographic bony union, and had undergone removal of plate were included in this study. All patients had undergone surgical fixation using superior plating for the clavicle fracture. The criteria for radiographic union were (1) a bridging callus formation or complete obliteration of the gap between fracture fragments, (2) no further migration of the fixation construct and no fracture displacement, and (3) pain-free. All patients included in this study met the criteria of radiographic union. The patients that were excluded were patients with fixation methods other than plate (e.g., Knowles pins) (*n* = 80), fracture nonunion (*n* = 21), loss of reduction that required refixation (*n* = 21), surgical site infection (*n* = 4), periprosthetic fracture that required refixation (*n* = 2), and further traumatic events that required a second operation (*n* = 0). Finally, a total of 278 patients were included in this study (Fig. 1).

During surgery, the fracture site was examined after the plate and screws were removed to assure stable and solid union of the fracture. After the surgery, the patients were allowed a full range of motion without restrictions. The operated arm was not allowed to perform weight-bearing activities during the first month after the surgery. After the first month, the patient was allowed to perform weight-bearing activities as tolerated.

**Fig. 2:**
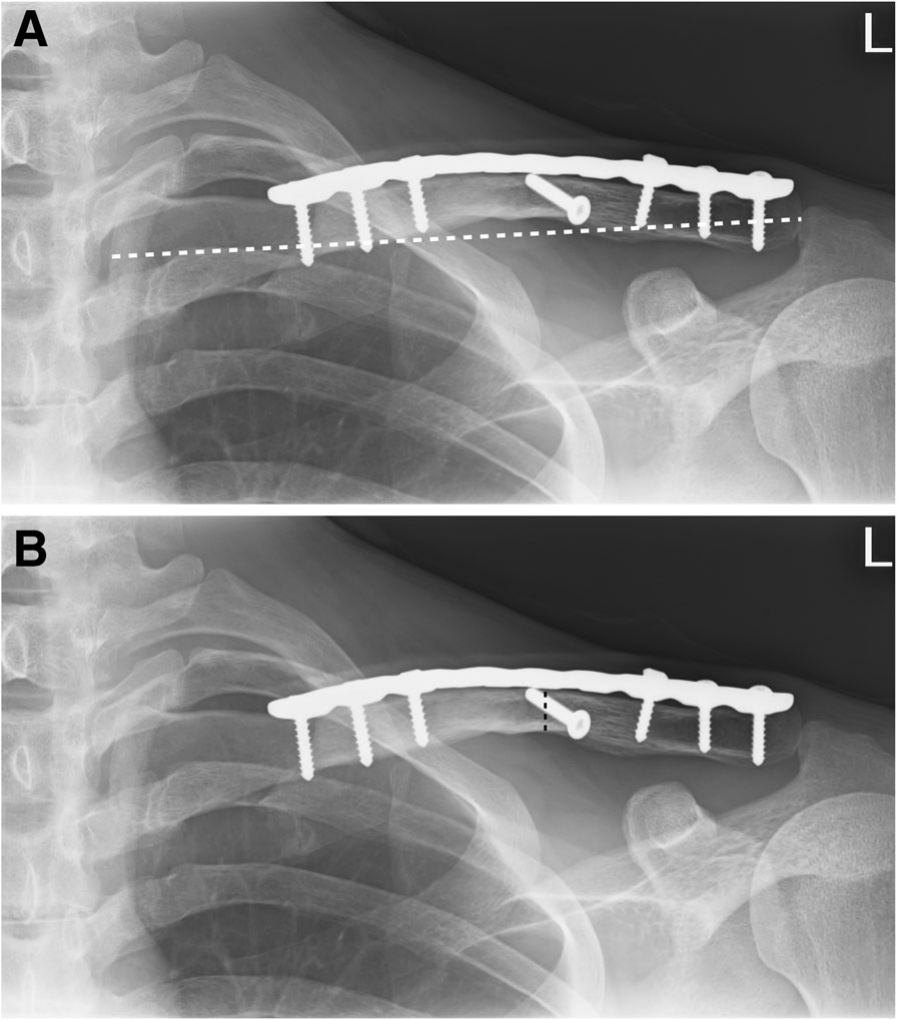
**a** On postoperative anteroposterior projection of clavicle, clavicular length (white dashed line) is defined as the length between the midpoints of both ends of the clavicle. **b** On a postoperative anteroposterior projection of clavicle, the bone diameter at the fracture site (*black dashed line*) is defined as the smallest diameter perpendicular to the plate at the fracture site

Standard clavicle anteroposterior projection was used for serial radiographic assessment. Plain X-rays taken immediately after the surgery were reviewed to measure postoperative “clavicular length” and “bone diameter at the fracture site.” “Clavicular length” was defined as the length between the midpoints of both ends of the clavicle (Fig. 2a). Bone diameter at the fracture site was defined as the smallest diameter that was perpendicular to the plate at the fracture site (Fig. 2b). In addition, callus formation was recorded on postoperative images.

SPSS 17.0 (SPSS Inc., Chicago, IL, USA) was used for all data analysis. Data are presented as mean, range, and standard deviation (SD) for continuous variables and as number (%) for categorical variables. Fisher’s exact test was used to compare differences between the two groups for each discrete variable when one or more of the cells in the contingency table had an expected frequency of < 5. Student’s *t* test was used to compare differences between the groups for each continuous variable. Receiver operating characteristics (ROC) curve analysis was used to validate the area under the curve (AUC) and arrive at an optimal cutoff value to predict refracture. Variables with *p* value < 0.05 in univariate analyses were entered into a multivariate logistic regression model. A forward stepwise method was used to determine independent risk factors. The results are expressed as adjusted odds ratio (aOR) plus a 95% confidence interval (CI). A *p* value < 0.05 was considered statistically significant.

## Results

We reviewed the records of 278 patients: men = 190 (68.3%), women = 88 (31.7%); mean age 40.1 ± 15.1 years; mean height 167.2 ± 9.0 cm; mean weight 68.3 ± 14.3 kg; mean body mass index (BMI) 24.2 ± 0.39; and mean interval between index fixation and plate removal 12.5 ± 7.5 months (range 3–71 months). All patients were divided into two groups: (a) with refracture after plate removal (20 patients) and (b) without refracture (258 patients with uneventful recovery).

**Fig. 3:**
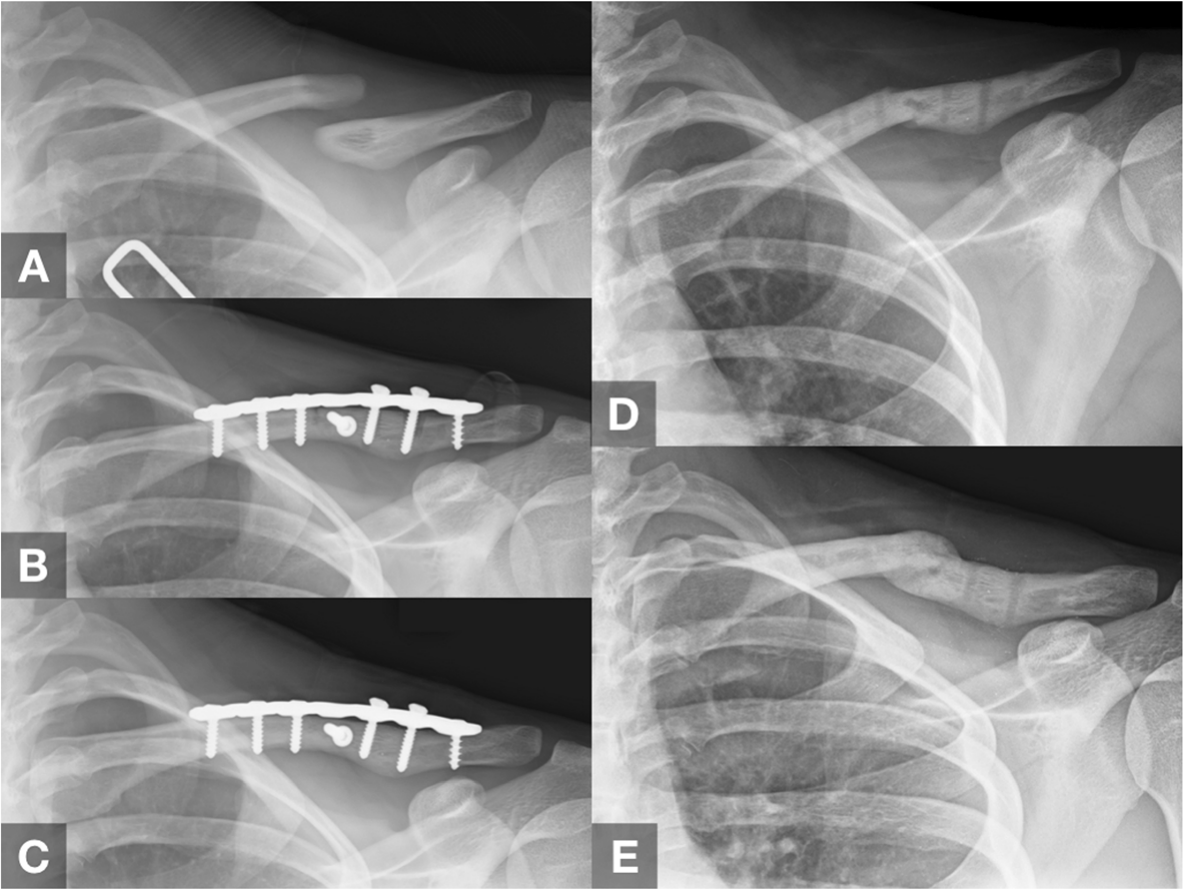
Case demonstration of a 25-year-old female patient. **a** Preoperative anteroposterior radiograph of the clavicle. **b** Immediately after open reduction and plate fixation surgery. **c** Twelve months after fixation surgery, before plate removal. **d** Refracture occurred 4 weeks after plate removal. **e** Conservative treatment for 3 months with solid radiographic union

The refracture rate was 7.2% (*N* = 20). After the plate was removed, none of these patients had experienced additional trauma events (e.g., a fall, a direct blow, or a sprain) but felt sudden pain and disability during activities of daily life (e.g., put on clothes, reach out for things, or take a bath). Nineteen patients had a fracture at the previous fracture site, and one was at a screw hole. The mean interval between plate removal and refracture was 23.9 days (range 1–84 days) (Table 1). Eleven patients (55%) were treated conservatively with an arm sling. Six patients reached solid union in a mean of 4.3 months (range 3–7 months) (Fig. 3). One patient showed callus formation but not solid union. The remaining four patients showed no further callus formation and no further fracture displacement; therefore, they underwent no further surgical fixation. Nine patients (45%) had undergone refixation with a plate, and all achieved radiographic union. Four patients underwent subsequent plate removal surgery without a refracture. The remaining five patients did not undergo subsequent plate removal surgeries.

**Table 1:** Patient demographic data

	Refracture (*n* = 20)	No refracture (*n* = 258)	*p*
Sex (*n* [%])			< 0.001
Male	6 (30%)	184 (71.3%)	
Female	14 (70%)	74 (28.7%)	
Age (years)	42.8 ± 13.1 (25–67)	39.9 ± 15.3 (24–84)	0.363
Body height (cm)	162.9 ± 9.8 (148–181)	166.9 ± 13.7 (142–189)	0.107
Body weight (kg)	57.7 ± 14.5 (38–91)	69.1 ± 14.0 (42–135)	0.003
Body mass index	21.4 ± 3.0 (15.3–28.4)	24.5 ± 3.9 (16.1–38.8)	< 0.001
Interval between fixation and removal (months)	11.6 ± 8.6 (3–44)	12.6 ± 7.3 (3–71)	0.621
Interval stratification (*n* [%])			
< 12 months	12 (60%)	114 (44.2%)	0.128
≥ 12 months	8 (40%)	144 (55.8%)	
< 18 months	18 (90%)	218 (84.5%)	0.392
≥ 18 months	2 (10%)	40 (15.5%)	
< 24 months	19 (95%)	240 (93.0%)	0.595
≥ 24 months	1 (5%)	18 (7.0%)	
Refracture: days after plate removal	23.9 ± 23.5 (1–84)	–	

All values are mean ± standard deviation (range) or *n* (%)

The refracture group contained significantly more women than the non-refracture group (70% vs. 28.7%; *p* <  0.001); its members weighed significantly less (mean 57.7 ± 14.5 vs. 69.1 ± 14.0 kg; *p* = 0.003) and had a significantly lower mean BMI (21.4 ± 3.0 vs. 24.5 ± 3.9 *p* <  0.001) than the non-refracture group. Age, height, and interval between fixation and plate removal were not significantly different (Table 1).

A dynamic compression plate was the fixation device for the index surgery in 261 (93.8%) patients (Table 2). Wire was used in 56 (20.1%) patients, and interfragmentary screws were used in 98 (35.3%). The most common fracture patterns were AO/OTA 15.2B (wedge fracture: *n* = 204, 73.4%) and Robinson type 2B1 (simple or wedge comminuted, displaced fracture: *n* = 201, 72.3%). The choice of fixation device, use of wires, use of interfragmentary screws, AO/OTA classification, Robinson fracture classification, and the presence of callus formation were not significantly different between the groups. Compared with patients without a refracture, clavicular length was significantly shorter (mean 15.0 ± 1.4 vs. 16.1 ± 1.4 mm, *p* = 0.002), and bone diameter at the fracture site was smaller (mean 1.14 ± 0.23 vs. 1.25 ± 0.19 mm, *p* = 0.021) in the refracture group.

**Table 2:** Radiographic assessment of the fractures

	Refracture (*n* = 20)	No refracture (*n* = 258)	*p*
Fixation device			0.270
Dynamic compression plate	20 (100%)	241 (93.4%)	
Locking compression plate	0	16 (6.6%)	
Wires used?			0.193
Yes	6 (30%)	50 (19.4%)	
No	14 (70%)	208 (80.6%)	
Interfragmentary screws used?			0.154
Yes	4 (20%)	94 (36.4%)	
No	16 (80%)	164 (63.6%)	
AO/OTA classification			0.965
15.2A	3 (15%)	38 (14.7%)	
15.2B	15 (75%)	189 (73.3%)	
15.2C	2 (10%)	31 (12.0%)	
Robinson classification			0.910
2A1	0	2 (0.8%)	
2A2	1 (5%)	23 (8.9%)	
2B1	15 (75%)	186 (72.1%)	
2B2	4 (20%)	47 (18.2%)	
Clavicular length (mm)	15.0 ± 1.4 (12.9–17.5)	16.1 ± 1.4 (12.1–19.3)	0.002
Bone diameter at the fracture site (mm)	1.14 ± 0.23 (0.81–1.79)	1.25 ± 0.19 (0.73–1.64)	0.021
Callus formation			0.567
Yes	1 (5%)	10 (3.9%)	
No	19 (95%)	248 (96.1%)	

All values are mean ± standard deviation (range) or *n* (%)

A multivariate logistic regression model showed that the following factors were significantly related to refracture: female (aOR 4.74; 95% CI 1.6–14.1) and lower BMI (with every 1-unit decrease, aOR 1.25; 95% CI 1.06–1.48) (Table 3).

**Table 3:** Risk factors for middle third clavicle refracture after removing the plate in multivariate logistic regression analysis

Factors	aOR	95% CI	*p*
Female	4.74	1.6–14.1	0.005
BMI (for every 1-unit decrease)	1.25	1.06–1.48	0.008

*aOR* adjusted odds ratio, *CI* confidence interval, *BMI* body mass index

**Fig. 4:**
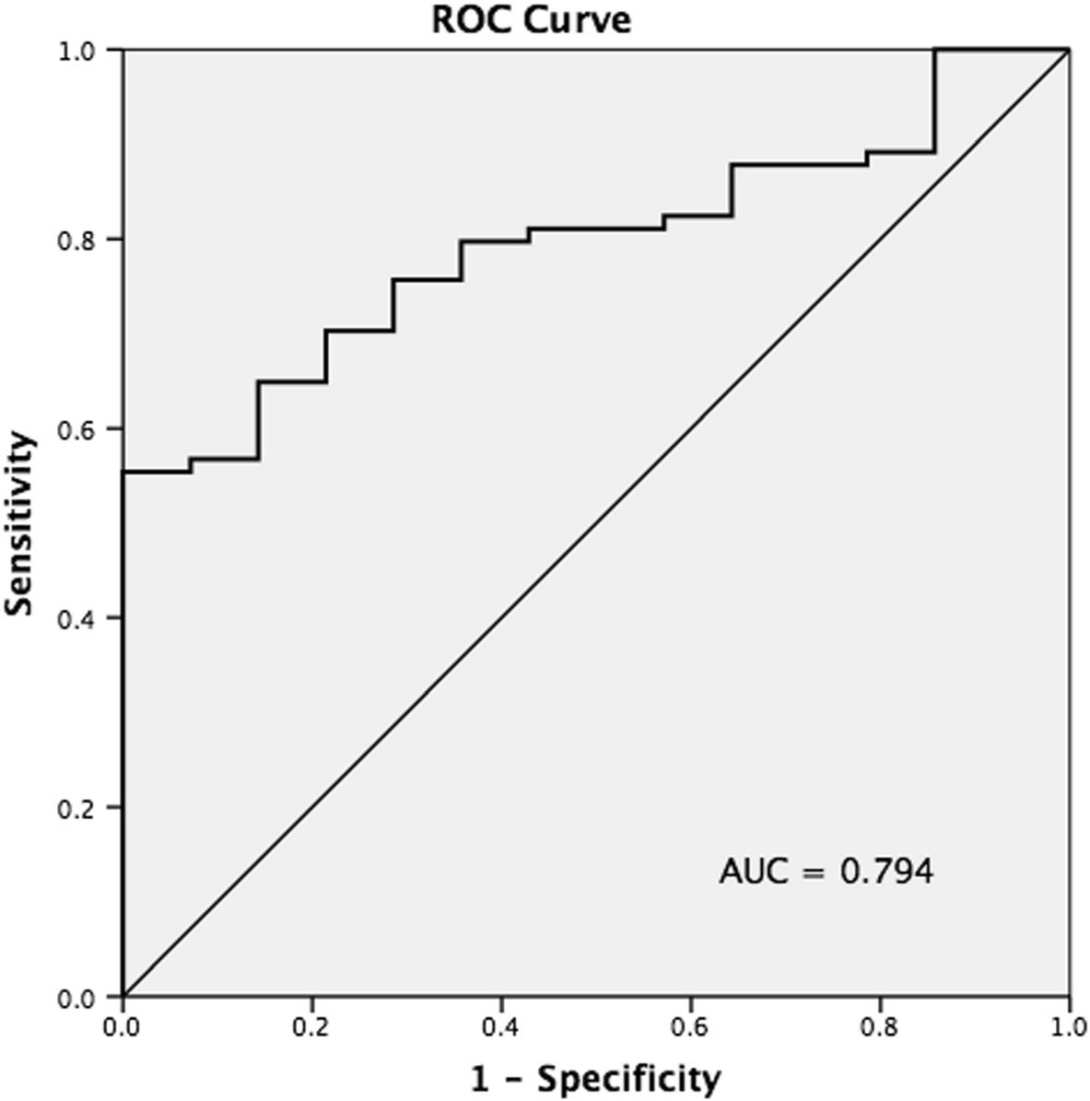
Receiver operating characteristic (ROC) curve to determine refracture events. Area under the curve based on BMI was 0.794 (95% CI 0.696–0.893). Optimal BMI cutoff value was 22.73

A subgroup analysis of the 88 female patients showed that 14 patients had a refracture and 74 did not (Table 4). Patients in the refracture group weighed less (mean 50.5 ± 8.2 vs. 58.7 ± 1.1 kg) and had a lower mean BMI (20.0 ± 2.0 vs. 23.6 ± 4.1). Mean age, interval between fixation and plate removal, patients with menopause, and all other radiographic parameters were not significantly different. A multivariate regression analysis showed that BMI was the only significant risk factor for refracture (with every 1-unit decrease, aOR = 1.52; 95% CI 1.16–2.00). Using ROC curve analysis, the AUC based on BMI was 0.794 (*p* = 0.001). The optimal cutoff value was 22.73 (sensitivity 55.4%, specificity 100%, and accuracy 62.5%). (Fig. 4).

**Table 4:** Subgroup analysis of the 88 female patients

	Refracture (*n* = 14)	No refracture (*n* = 74)	*p*
Age (years)	43.0 ± 15.5 (25–67)	44.7 ± 17.8 (15–78)	0.739
Menopause	5 (35.7%)	36 (48.6%)	0.277
Body height (cm)	158.4 ± 7.3 (148–175)	157.5 ± 6.1 (141.5–170)	0.621
Body weight (kg)	50.5 ± 8.2 (37.8–64)	58.7 ± 11.1 (42.4–107.2)	0.010
Body mass index	20.0 ± 2.0 (15.3–20.9)	23.6 ± 4.1 (17.6–38.8)	0.002
Interval between fixation and removal (months)	13.3 ± 9.7 (5–44)	12.2 ± 5.8 (4–36)	0.575
Interval stratification			0.223
< 12 months	6 (42.9%)	43 (58.1%)	
≥ 12 months	8 (57.1%)	31 (41.9%)	

All values are mean ± standard deviation (range) or *n* (%)

## Discussion

In this study, the overall refracture rate after plate removal was 7.2%, which is slightly higher than the 1~5% reported in current literature [9–11]. Our study might be the first to validate risk factors for refracture after plate removal in patients with midshaft clavicle fracture. Female patients and a lower BMI were risk factors associated with higher refracture rates. Therefore, removing the plate in a female patient with a low BMI requires a thorough evaluation.

The incidence rate of reoperation after surgical treatment for clavicle fractures ranges from 18.6 to 74% [5–8, 12, 13]. Hardware irritation was the most common reason for removing the plate (incidence rate 8–50%) [5–8, 14]. One study [5] (143 patients) reported that 29 (20%) patients required reoperations with implant irritation (*n* = 25) being the most common reason, followed by implant failure (*n* = 2) and fracture nonunion (*n* = 2). Another study [6] (153 patients) reported a reoperation rate of 38% (*n* = 58), primarily because of isolated plate removal (*n* = 42, 72%). The significant risk factors for isolated plate removal were using a non-precontoured plate and body height < 175 cm. The overall reoperation rate of a study [7] of 1350 patients was 24.6% (*n* = 332) with isolated plate removal (*n* = 254, 18.8%) being the most frequent reason. Female was a risk factor of reoperation with an aOR of 1.7. A study [8] of 56 patients reported a high plate-related irritation rate (*n* = 39 [70%]), and the plate removal rate was 50%. The authors concluded that both plate and intramedullary fixation were frequently associated with implant-related irritation that resulted in high rates of implant removal.

### Factors of refracture: patient demographics

In the current literature, risk factors for isolated plate removal for midshaft clavicle fracture include female, body height < 175 cm, and using a non-precontoured plate [5–7]. Some authors hypothesized that skin irritation secondary to supportive undergarments that cross the clavicle plate might explain why female was a risk factor for implant removal [7]. Female and a lower BMI were risk factors for refracture in our study. In the Framingham study (women 693; men 493; mean age 76 years old) [15], authors determined an association between BMI and bone mineral density (BMD). Postmenopausal osteoporosis in women also results in lower BMD, which leads to a greater risk of vertebral and non-vertebral fractures; the clavicle, however, was not a common site [16]. Our study population was relatively young: mean age (all patients) 40.1 years old, mean age (all women) 44.5 years old. Only 44 of our 88 women (50%) were in menopause, but that was not a risk factor for refracture. Because the clavicle is not a common site of osteoporotic fracture and because our study population was relatively young, a BMD examination was not routinely done for assessment.

Female was a risk factor for both implant removal after a clavicle fracture [5, 7] and for refracture after removing the implant. The refracture rate in our female patients was as high as 15.9% (*n* = 14/88), but only ~ 3.2% in male patients (*n* = 6/190). BMI was another risk factor for refracture. With every 1-unit decrease in BMI, the aOR of refracture increased by 1.25 overall and increased by 1.52 for women. Using ROC curve analysis of BMI in women, the optimal cutoff value was 22.73. The refracture rate of women with a BMI < 22.73 was as high as 29.8%. The mean duration from removal to refracture in the 14 women with a refracture was 19.1 days. None of the patients that had a refracture had a major traumatic event (e.g., a fall or a direct force) as all refractures occurred during regular daily activities. Our standard procedure is to restrict the operated arm from weight-bearing for 1 month. Despite this strict protocol, there were still two patients who had a refracture after 1 month. Thus, an extended period of protection beyond the first month may not be necessary because bone union was radiographically confirmed before the plate was removed. With the results noted in these studies, removal of hardware in a female patient with a low BMI is associated with higher refracture rates and thorough assessment prior to removal of hardware is essential.

### Factors of refracture: radiographic parameters

To our knowledge, there are no current studies that discussed the risk factors associated with refracture of a clavicle after the plate has been removed. However, several reports have discussed the risk factors for refracture in forearm bones, which are about the same size as the clavicle. Failure to achieve adequate compression during surgery and residual bone defects that have not been adequately managed were risk factors of refracture after removing an implant [17]. A more comminuted fracture pattern and residual bone defect because of absorbed fragments were associated with refracture [18]. We used both the AO/OTA and the Robinson classifications to compare fracture patterns and comminution between groups. With a wedge or a multi-fragmented fracture pattern, a wire or an interfragmentary screw might be necessary for anatomic fracture reduction. We found that none of those parameters was associated with a refracture.

Removing the cortical screws might lead to a microfracture near a screw hole, which will increase stress [19]. Loss of bone strength, both bending and torsional strength, was proportional to screw hole diameter and inversely proportional to bone diameter. A ratio of screw diameter to bone diameter of 0.25 leads to a 40% decrease of bone strength [20, 21]. In a Chinese population with a mean age of 37 years old, a computed tomography (CT) scan showed that the diameter of the clavicle at the sternal and acromial ends, and in the middle shaft, were greater in males than in females [22]. Based on these studies, removing a screw from a long bone segment (e.g., the midshaft clavicle) with a smaller diameter in a female might lead to a greater reduction in bone strength, which might be associated with refracture. However, only one patient had a refracture at a screw hole. Bone diameters were smaller at the fracture site, and the clavicles were shorter in the refracture group, but neither factor was significant in a multivariate analysis. Female might be a confounder for these two parameters according to a morphometric analysis [22].

Dynamic compression plates were used in 261 (93.9%) patients. Most were anatomically reduced and had achieved absolute stability using lag screws and dynamic compression plates. That might explain why there was no callus formation in most patients (*n* = 267, 96.0%). Under these circumstances, no further fracture displacement or migration of the fixation construct are the only signs that indicate radiographic union. However, 19 patients who met these criteria but had a refracture after the plate had been removed. No radiographic parameters on the clavicle anteroposterior projection were associated with refracture in our study. Additional X-ray projections or CT scans might be helpful, but they require additional costs and, because of the low incidence rate of refracture, are not practical to add to routine examinations.

This study has some limitations. First, the overall refracture rate (7.2%) was higher than the incidence rate reported in the literature: range 1–5% [9–11]. The mean interval between index fixation and plate removal was 12.5 months (not significantly different between groups). Some authors have recommended that the interval between index fixation and implant removal in the upper extremities should be longer than 18 months [18]; however, there is a lack of high level of evidence for this suggestion. Although we found no association between the interval and refracture, the refracture rate might be lower if we extended the interval between the index surgery and removal of implant. Second, a BMD examination was not routinely done which is also important for fracture union. However, since our study population consisted mostly of healthy, younger patients, bone density should not be a critical issue. Third, we did not routinely perform CT to assess bony union before plate removal. However, the use of CT might be limited by artifact results from metallic implants that obscure details in the fracture site.

## Conclusions

The refracture rate was low after the plate had been removed from a midshaft clavicle fracture. On the other hand, there were no significant radiographic parameters on clavicle anteroposterior projection associated with refracture. In conclusion, plate removal after bone union of a midshaft clavicle fracture in female patients with a low BMI should be carefully evaluated and may lead to a higher risk of refracture after the surgery.

## Data Availability

The datasets used and/or analyzed during the current study are available from the corresponding author on reasonable request.

## Abbreviations

AUC: Area under the curve
BMD: Bone mineral density
BMI: Body mass index
CI: Confidence interval
CT: Computed tomography
OR: Odds ratio
ORIF: Open reduction and internal fixation
ROC: Receiver operating characteristics
SD: Standard deviation
